# Generating Light from Upper Excited Triplet States: A Contribution to the Indirect Singlet Yield of a Polymer OLED, Helping to Exceed the 25% Singlet Exciton Limit

**DOI:** 10.1002/advs.201500221

**Published:** 2015-12-12

**Authors:** Vygintas Jankus, Murat Aydemir, Fernando B. Dias, Andrew P. Monkman

**Affiliations:** ^1^ OEM Research GroupPhysics DepartmentUniversity of DurhamSouth RoadDurhamDH1 3LEUK

**Keywords:** charge transfer states, delayed fluorescence, OLED, reverse intersystem crossing, triplet fusion, triplet–triplet annihilation

## Abstract

The mechanisms by which light is generated in an organic light emitting diode have slowly been elucidated over the last ten years. The role of triplet annihilation has demonstrated how the “spin statistical limit” can be surpassed, but it cannot account for all light produced in the most efficient devices. Here, a further mechanism is demonstrated by which upper excited triplet states can also contribute to indirect singlet production and delayed fluorescence. Since in a device the population of these T_N_ states is large, this indirect radiative decay channel can contribute a sizeable fraction of the total emission measured from a device. The role of intra‐ and interchain charge transfer states is critical in underpinning this mechanism.

## 1 Introduction

From the outset of research into organic light emitting diodes (OLEDs) it has been assumed that the process of charge recombination which generates excitons is controlled by random spin statistics with singlet and triplet excitons formed in the ratio 1:3. Wohlgenannt et al. questioned this and by measurements of singlet and triplet formation cross sections concluded that the recombination process was in fact spin dependent in polymers.^1^ This first result stimulated many experimental and theoretical studies of possible physical mechanisms that would give rise to spin dependent recombination.^2^ However, more recent studies have shown that the process of triplet–triplet annihilation (TTA) and the production of singlets from TTA, by triplet fusion (TF),^3^, ^4^, ^5^, ^6^ contributes to the secondary production of singlet excitons. The initial recombination process still obeys spin statistics whilst the total singlet production yield can exceed 25%. TTA is key to our understanding of triplet exciton dynamics in organic materials and therefore essential for a proper understanding of the use of organic materials in many applications. However, the efficiency of TF has not been directly measured, especially in the luminescent materials used in OLEDs. Given that in an OLED very high triplet populations are generated in relatively well confined, thin recombination layers, typical 5–10 nm,^7^ the triplet density will be very high and TTA very efficient. Thus, TF will contribute to the number of singlets produced in the device, and this contribution will depend critically on the TF branching ratio in TTA. Jortner et al.^8^ first proposed that quantum spin statistics controls the process of singlet formation arising from the annihilation of two triplet excitons


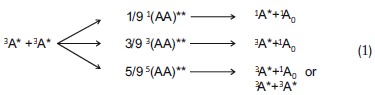


where ^1^A^*^, ^3^A^*^, and ^5^A^*^ are excited singlet, triplet, and quintuplet states and ^1^(AA)^*^, ^3^(AA)^*,5^, (AA)^*^ are interaction pairs with singlet, triplet, and quintuplet spin multiplicities. The subsequent decay of the triplet pairs yields an excited triplet exciton and a ground state molecule. In this scenario only 5.5% singlet states will be produced. However, evidence from OLED devices and TTA up‐conversion measurements suggest that this value is incorrect. For example, Cheng et al.^9^ have estimated a 0.125 singlet yield in their solution measurements. Kondakov et al. in detailed measurements of delayed electroluminescence from OLED's,^10^ indirectly implied that the contribution to the overall electroluminescence yield of their devices from TF can be 20%^11^ or 50%^12^ depending on the emissive material. This would require a far higher TF singlet yield than 0.055.

OLEDs are very complicated systems where charge transport, exciton migration, exciton polaron quenching and other processes take place. This makes estimation of TF efficiency from OLED measurements very difficult. Kondakov et al rationalized the 20% delayed fluorescence (DF) contribution from TF by pointing out that quintuplet interaction pairs cannot participate in TTA because they are energetically untenable, the energy of two triplet excitons is not enough to produce a quintuplet state,^13^ hence changing the dynamics described in Scheme I, and yielding a total singlet yield of 0.2, i.e., $\sum_{n=0}^{\infty }\;\frac{13^{n}}{18^{n}+1}$, taking into account triplet recycling, that is the T_N_ states produced by the triplet annihilation channel relax to T_1_ states, that can then annihilate again until the triplet reservoir is exhausted (see the Supporting Information for detailed scheme).

Indeed, in the seventies this had been shown experimentally in small organic molecules, for example, Groff et al. indirectly determined a 0.22 yield of TF to account for the delayed fluorescence in anthracene^14^ while Ern et al. found a very similar TF yield of 0.19 for pyrene dimers.^15^

**Figure 1:**
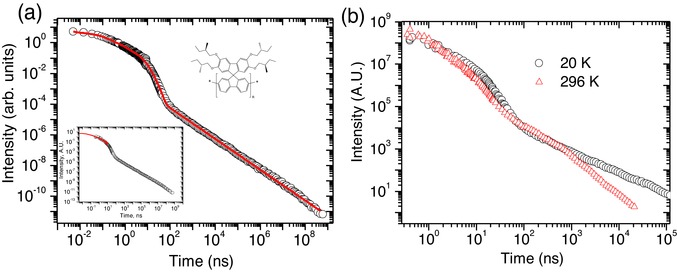
a) Emission decay of PSBF:zeonex spincoated film recorded at 20 K (black circles). The initial part of the decay (up to ≈20 ns) is assigned to prompt fluorescence, the long tail appearing as a power law (from 200 ns to 1s) is assigned to delayed fluorescence arising from triplet–triplet annihilation. An intermediate decay channel is found between 20 and 200 ns. The red line is a fit with Equation (2). The curve was obtained by combining decays recorded using nanosecond gated time resolved spectroscopy (from 1 ns to 1 s, at 15 μJ per pulse excitation) and singlet photon counting techniques (from 3 ps to 10 ns, at <1 nJ per pulse excitation). b) Emission decay of PSBF spincoated film recorded at 20 K (black circles) and 296 K (red circles).

In our direct measurement of the singlet to triplet exciton yield in OLED devices based on the polyspirobifluorene (PSBF) polymer, structure shown in **Figure** **1**, a total singlet yield of 0.44 was obtained.^16^ Further, King et al. have also shown unambiguously that DF accounts for up to 25% of the total EL output in other polymer devices^17^ from measurements of delayed electroluminescence, first observed by Sinha et al.^10^ Secondary singlet states generated by TF thus give rise to total singlet yields which exceed the classical spin statistical limit of 0.25 in both polymer and small molecule OLED devices, implying that charge recombination *does not have to* violate spin statistics in order to exceed an internal quantum efficiency of 25%.^18^ In order to understand the total secondary singlet production mechanism we set out to measure *directly* the efficiency that singlets are produced from triplets in PSBF. We subsequently discovered that two triplet channels are operative. As well as the normal channel of TF, a second channel is present. When triplets annihilate to generate the upper triplet excited state, T_N_, these upper triplet states can also generate singlet states by decaying via a charge transfer state. The impact this mechanism has on the overall efficiency of light generation is profound and explains the high efficiency of many polymer and small molecule fluorescent OLED emitters.

## 2 Results

To measure TF yield in films directly, we have developed a method using nanosecond gated time resolved spectroscopy similar to that which we use to study triplet exciton dynamics in polymer films, see refs. 4 and 6 for details.

**Figure 2:**
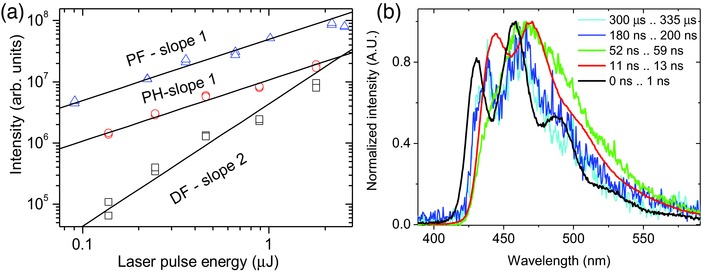
a) “Prompt” fluorescence (PF recorded 3 ns..–13 ns), phosphorescence (PH‐100 μs .. 5 ms), and delayed fluorescence (DF‐100 μs..5 ms) dependence on excitation energy from PSBF film recorded at 20 K. The slope of the DF power dependence being <2 indicates mixed linear and quandratic contributions to the total decay. b) Time evolution of emission from the PSBF spincoated film at 20 K. Initial vibronically resolved S_1_^1^(π, π*) emission is observed in the first nanosecond (black trace), spectral diffusion of singlet excitons red shifts this S_1_ emission over ≈10 ns (red trace) and concomitant with the appearance of increased red edge emission. By 50 ns, this red unstructured emission dominates ascribed to intramolecular CT states.^22^ After 200 ns, and on into the microsecond regime delayed S_1_^1^(π, π*) again observed ascribed to TF. The CT state is ≈0.1 eV lower in energy than the S_1_.

The key element in these measurements is the ability to capture both prompt and delayed fluorescence simultaneously (one curve) as shown in Figure 1. We ascribe the decay up to ≈20 ns to prompt fluorescence (PF). This is confirmed by TCSPC measurements on the same films giving an average fluorescence lifetime of PSBF of ≈3 ns.^19^, ^20^ More detailed analysis reveals a triexpontential decay with two major emitting species, the initially photogenerated local exciton, ^1^LE (≈1 ns), and a slightly lower energy ^1^CT state (≈5 ns).^21^ The decay tail appearing from ≈200 ns to 1 s as a power law is assigned to DF. This DF appears in PSBF as a result of TF, as clearly shown in **Figure** **2**a—the DF intensity follows predominantly a quadratic dependence with increasing laser pulse intensity.

Given that PF follows an exponential decay law and DF a power law at 20 K, we can fit the luminescence decay curve using the equation

(2) $$y\;=\;\sum_{N=1}^{k}A_{N}\exp(-x/t_{N})\;+\;Ax^{t}$$

More than one exponential term is needed, as PF decay in PSBF films at 20 K is not mono exponential as shown in previous detailed fluorescence lifetime studies.^19^, ^20^ Integration of the exponential terms gives the PF fraction of the total emission and integration of the power law term gives the DF contributions, ϕ_DF_ of the total sample emission as in Equation (3), see the Supporting Information for details.

(3) $$\varphi_{\mathrm{DF}}\;=\;\frac{R}{\left(1\;+\;R\right)\varphi_{T}}$$

Using Equation (3), we determined *ϕ*_DF_ at 20 K to be 0.33 ± 0.08 and at room temperature to be 0.14 ± 0.04, see Supporting Information for details. At room temperature, and at intermediate times (≈20–200 ns, Figure 1b) the DF intensity makes a larger contribution in comparison with the DF at 20 K over the same time period, indicating an increase in the thermally activated DF process. However at ≈800 ns the DF undergoes a rapid intensity roll‐off and overall DF efficiency decreases substantially. This DF intensity roll‐off is due to a change from dispersive to nondispersive triplet exciton migration and has been observed previously in both small molecule (*N*,*N*′‐diphenyl‐*N*,*N*′‐bis(1‐naphthyl)‐1,1′‐biphenyl‐4,4″‐diamine)^6^ and polymer (polyfluorene)^3^, ^4^ films. At room temperature, nonradiative decay becomes much stronger (in polymers) and so the NR quenching of the 3LE triplet states competes with TF reducing the overall amount of singlets generated by TF and so the overall DF yield reduces.

Turning to our previous PSBF OLED device data with measured singlet yield at 20 K of 0.44 ± 0.04.^16^ Taking ϕ_DF_ = 0.33 at 20 K, and a spin independent charge recombination triplet yield of 75%, then the contribution of DF to the total singlet yield would be 0.33 × 0.75 = 0.25 ± 0.06. 0.25 ± 0.06 added to the directly created singlet excitons (with 0.25 yield) gives a total singlet production yield of 0.50 ± 0.06 which is within error limits is in excellent agreement with the measured 0.44 ± 0.04. The agreement is striking especially bearing in mind that very different experimental methods were used to determine them.

However, as pointed out above, for PSBF it is not possible to achieve ϕ_DF_ > 0.2 from TF alone. Clearly, in PSBF 2*E*_T_>*E*_Tn_ (*E*_T_ = 2.22 eV, *E*_Tn_ = 3.77 eV)^16^ so that the maximum ϕ_DF_ can only be 0.2. As our measurements require that ϕ_DF_ > 0.2 we conclude that there must be a further contribution to DF. One possible process could be upper excited state reverse intersystem crossing from the T_N_ state (uRISC), i.e., from T_N_ → S_1_. This process would be in competition with internal conversion of T_N_ to lower energy triplet states. However, it has never before been observed in luminescent polymers, but T_N_ → S_N_ transitions have been reported in a few organic molecules.^23^, ^24^, ^25^ If uRISC where to be this source of the extra singlets, then it would require a contribution of 0.13 to the total DF emission. This requires an overall triplet to singlet yield (including T_N_ → S_1_ uRISC) of 0.33, that is 80% of triplets annihilate via the triplet channel producing T_N_ states with 0.4 yield (two T_1_ states required to produce one T_N_ state), and therefore, 0.13/0.4 = 0.33 triplets need to decay by uRISK to generate the required DF singlets. There are reports of uRISC yields of 0.17 in dibromoanthracene,^26^ ≈0.70 in cyanine dyes,^27^ 0.80 in rose bengal dye^23^ and 0.90 in tetraphenylphorphyrin.^23^ However, we do not believe this to be the case here from the dynamics of the process we subsequently measure.

**Figure 3:**
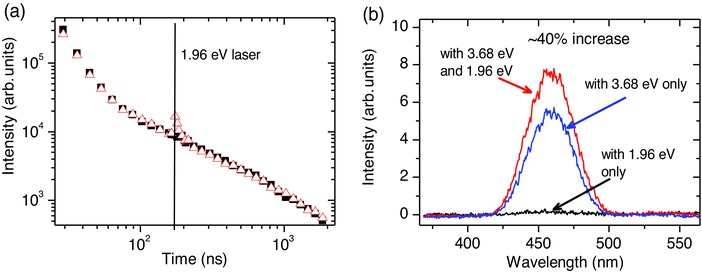
a) Decay of 0.01% PSBF in zeonex at 20 K when exciting with 3.68 eV at 0 time only (full squares) and when exciting at 3.68 eV at 0 time *and* at 1.96 eV at 177 ns (empty triangles, zoomed in the inset). Straight line denotes the excitation time of 177 ns with 1.96 eV pulsed laser. b) Time resolved spectra recorded from 178 ns..208 ns with 3.68 eV excitation at 0 time only, with 3.68 eV excitation at 0 time and 1.96 eV excitation at 177 ns, and with 1.96 eV excitation only (indicated appropriately). Single band pass interference filter was used to record emission signal.

Careful analysis of the DF spectra, **Figure** **3**, clearly shows that the delayed emission is complex and evolves with time. From the initial S_1_^1^(π, π*) decay, i.e., vibronically resolved emission measured in the first nanosecond, we observe a spectral red shift with time and loss of vibronic resolution. By 50 ns, this red‐shifted unstructured emission dominates. We have previously shown that this arises from decay of intramolecular CT states on the PSBF chain. The PSBF ^1^CT state is ≈0.1 eV lower in energy than the S_1_.^21^, ^22^ After 200 ns, and on into the microsecond regime we again observed the re‐emergence of structured, delayed S_1_^1^(π, π*), ascribed to TF.

As has recently been shown, triplet states can be efficiently harvested if they couple to energetically close lying triplet states, yielding thermally activated delayed fluorescence (TADF). A very small ^1^CT–^3^CT gap occurs in charge transfer states in spiro materials because of the orthogonal nature of highest occupied molecular orbital (HOMO) and lowest unoccupied molecular orbital (LUMO) states, where negligible orbital overlap yields very small electron exchange energy.^28^ This mechanism can be 100% efficient with a small enough CT splitting^29^ and in OLEDs produces very efficient devices through this TADF triplet harvesting mechanism.^30^, ^31^ Therefore, in PSBF at intermediate times, ^1^CT emission arising from both initially created ^1^CT states (via electron transfer from the excitonic S_1_ excited state) and TADF could be expected. The back electron transfer rate ^3^CT →^3^LE to the very low lying PSBF ^3^(π, π*) ^3^LE state is typically relatively slow (≈5 × 10^6^ s^−1^)^23^ which will allow ^3^CT harvesting to compete with this IC channel, giving rise to the observed ^1^CT emission at intermediate times but only TF at longer times. The increase observed in the PSBF CT emission contribution at room temperature compared to 20 K^21^ clearly indicates that the TADF recycling^28^ of ^3^CT to ^1^CT is active in the PSBF. Because TF does not involve any electron transfer, TF must regenerate ^1^LE not ^1^CT singlet states.

To further explore how the DF from the CT state arises, 0.01% PSBF matrix isolated in zeonex films where studied at 20 K using a two pulse pump method (Figure 3). At this low polymer concentration, interchain TTA should be prevented and with a triplet yield of ϕ_T_ = 0.12,^20^ at low excitation intensities intrachain TTA should also be very low. Films were initially excited at 3.68 eV (S_0_ to S_1_) and the luminescence decay recorded (Figure 3a full squares). 177 ns after the initial (3.68 eV) excitation we excite with a second 1.96 eV pulse (Figure 3a empty triangles). 1.96 eV is not absorbed by the ground state^16^ and neither S_1_ states given that the lifetime of S_1_ is ≈1 ns,^19^ but rather excites the long lived T_1_ excitons to the higher T_N_ level, through an optically allowed transition.^20^ It is evident from the data in Figure 3a that after excitation with the 1.96 eV pulse, the DF intensity increases, by as much as 40%, Figure 3b. Comparable results have also been obtained using 1.55 eV pulses which are resonant with the allowed T_1_ to T_N_ transition, onset at 1.35 eV.^20^

Further, we were able to recorded time resolved emission spectra from 178 to 208 ns (relative to the initial 3.68 eV excitation) with three types of excitation. First, we excited with 1.96 eV (30–60 μJ per pulse) only to ascertain that there is no nonlinear two photon induced fluorescence (Figure 3b). We then excited with 3.68 eV (60–100 μJ per pulse) only to measure the base line delayed emission signal. Finally we excited with 3.68 and 1.96 eV at 177 ns delay to induce T_1_–T_N_ transitions. In the latter case we observed the intensity increase of ≈40% which indicates a relatively efficient increase in DF. We estimate from Figure 3a that the lifetime of this *induced* delayed emission to be, ≈5–10 ns, which is considerably longer than the 1 ns 1.96 eV pump pulse and much longer than the expected T_N_ state lifetime. This we believe rules out uRISK. Further, we clearly observe an increase in total DF, simply by exciting the T_1_ states to the T_N_ states, but the second excitation beam does not increase the total number of excited states, it only perturbs the triplet population (excites them). This second pump beam cannot induce extra TF, therefore a new physical decay channel must be accessed from the T_N_ population, i.e., a further radiative decay channel becomes operative from the T_N_ state.

## 3 Discussion

Given the lifetime and spectral shape of this induced delayed fluorescence, we ascribe it to radiative decay of the ^1^CT state. But, how does this state become populated from the T_N_? The T_N_ state will predominantly decay to the next lowest triplet state, not the lowest lying ^3^(π, π*) ^3^LE level but the (much closer in energy) ^3^CT triplet state, thus a kinetic competition will take place.^32^

A relatively long lifetime (>>100 ps) of the upper excited T_N_ state is not unreasonable given that we know for ‘polyfluorene’ backbone structures the T_N_ state is orthogonal to the T_1_ state^33^ and further T_N_, is 1.55 eV above T_1_. Given that the highest energy vibronic mode for PSBF is the 180 meV, C=C bond stretch, T_N,0_ lies at least 8 vibrational quanta above T_1,0_ giving rise to slow decay through the phonon bottle neck this causes.^34^ Indeed such a large T_N_–T_1_ energy gap can give rise to T_N_ fluorescence in the case where T_N_ lies below S_1_, showing that upper excited state lifetimes can be relatively long,^35^ at least on a par with S_1_ which readily allows S_1_ →^1^CT electron transfer. It can also be seen that the T_N_ state is ≈0.7 eV above ^3^CT giving a strong driving force for the electron transfer step.

**Figure 4:**
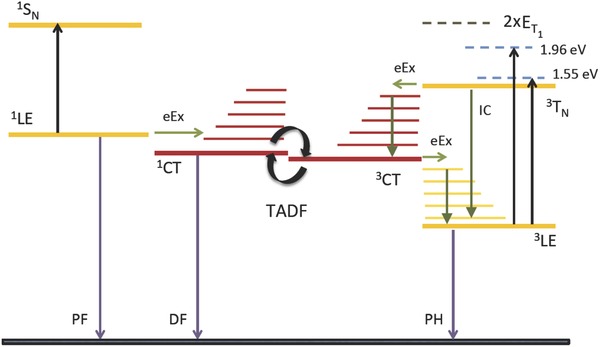
Schematic energy level diagram for PSBF constructed from fluorescence, phosphorescence, and photoinduced absorption measurements and delayed fluorescence, see ref. 16. From this Jablowski scheme the various decay channels for initial and photoinduced excited states can be seen in PSBF. The S_1_ state will be quenched by the ^1^CT state by electron transfer, ^1^CT can interconvert slowly by hyperfine coupling to ^3^CT. There is then a competition between TADF back to ^1^CT and ^3^CT quenching to the lowest energy ^3^LE state of the system.

Thus, a proportion of the T_N_ states created by TTA will decay via electron transfer to the ^3^CT state and via TADF generate the extra ^1^CT DF signal we observe after the second pump pulse. The ^1^CT–^3^CT gap in PSBF can be estimated from the previous results of King et al., who observed a temperature activation energy for DF in PSBF films of ≈14 meV.^36^ This was attributed to the gap between the ^1^CT state and the ^1^(π, π*) S_1_ state, however, this is too small for the gap measured spectroscopically, and in light of our recent findings on TADF, we can now understand this data as being a measure of the exchange energy in the CT manifold.^29^ The small exchange energy is comparable with other CT systems where there is near orthogonally between HOMO and LUMO level and is the case for the PSBF where we have previously shown that the charge transfer occurs across the orthogonal spiro unit, mediate by spiro conjugation and is in line with the results of Mehes et al. on spiro charge transfer molecules.^28^ This mechanism could readily give rise to an extra 0.13 contribution to the total DF. This then gives a new mechanism for generating DF from T_N_ states. As TTA produces a predominance of T_N_, then the presence of intermediate CT states that give efficient TADF gives efficient singlet production by this *induced* TADF mechanism. These competing decay channels are shown schematically in **Figure** **4**.

On reflection, this mechanism is the only way in which photoexcitation of T_N_ states (from T_1_ states) can give rise to extra DF as observed; if they simply decay back to T_1_ we would see no increase in DF after the second pulse. uRISK seems implausible as it would have been seem in many other, non‐CT containing luminescent polymers before, given the efficiencies required here and the lifetime of the T_N_ state we measure here.

## 4 Conclusions

We present the first direct evaluation of the total singlet exciton production yield from triplet excitons in a luminescent polymer, PSBF. We obtain a lower limit of ϕ_DF_ = 0.33 for total delayed fluorescence, which is clearly much greater than the “classical” TTA singlet TF yield of 0.055 and also above the expected 0.2 yield given that in the case of PSBF, only the quintuplet TTA channel is energetically unattainable. These results indicate that a further process must contribute to the efficiency of delayed fluorescence, which we identify as *induced* TADF via decay of T_N_ states to the ^3^CT state in competition with decay to the ^3^(π, π*) state. From our combined TF and induced TADF yield of 0.33 for PSBF, we achieve agreement with the total singlet production yield of 0.44 ± 0.04 determined for PSBF devices without breaking spin statistics in both recombination and TTA.

Our results show that triplet annihilation can generate singlets in two ways. First, through triplet fusion, where the encounter complex gains sufficient singlet wavefunction character that the two triplets become a singlet state. The second, distinct process requiring an intermediate charge transfer state. The normally wasted T_N_ state formed via the TTA triplet channel can decay by electron transfer, to ^3^CT which converts to ^1^CT through the TADF process. This mechanism then also produces photons through the radiative decay of the ^1^CT state.

More generally, many polymers either have intrachain CT states as is the case of PSBF, but also interchain CT states, or polaron pairs are also well known^37^ which are effectively exciplex states that also give rise to efficient TADF.^38^, ^39^ Thus, the T_N_ decay channel via the ^3^CT state can be an intrinsic mechanism for harvesting triplet states into singlets.^29^ Also, many CT small molecules which give mixed TADF and TTA, when the lowest lying ^3^LE state lies below the CT manifold still give good OLED performance. These molecules give a high triplet population leading to strong TTA and concomitant T_N_ population which could again be harvested by this induced TADF mechanism giving enhance singlet yields, but not 100% singlet production that true TADF systems.^32^, ^40^

## 5 Experimental Section

Solutions of PSBF where made in toluene at 10 mg mL^−1^ concentrations for spin coating. Guest host films in zeonex were prepared by drop casting from 0.01% PSBF in zeonex by weight onto sapphire substrates. Absorption and emission spectra were collected using a UV‐3600 double beam spectrophotometer (Shimadzu), and a Fluorolog fluorescence spectrometer (Jobin Yvon).

Phosphorescence, prompt fluorescence (PF), and delayed emission (DF) spectra and decays were recorded using nanosecond gated luminescence and lifetime measurements (from 400 ps to 1 s) using either a high energy pulsed Nd:YAG laser emitting at 355 nm (EKSPLA) or a N2 laser emitting at 337 nm. Emission was focused onto a spectrograph and detected on a sensitive gated iCCD camera (Stanford Computer Optics) having sub‐nanosecond resolution. PF/DF time resolved measurements were performed by exponentially increasing gate and delay times. Two pump measurements were made using the Nd:YAG laser as the master trigger source and via a delay generator a second diode laser was triggered after an appropriate time delay. Subsequent delayed emission was measured with the iCCD triggered by the master pulse with appropriate delay. Measurements were made at 20 K with samples in a displex cryostat.

## Supporting information

As a service to our authors and readers, this journal provides supporting information supplied by the authors. Such materials are peer reviewed and may be re‐organized for online delivery, but are not copy‐edited or typeset. Technical support issues arising from supporting information (other than missing files) should be addressed to the authors.

Supplementary
