# Supplementary material for: Generating Light from Upper Excited Triplet States: A Contribution to the Indirect Singlet Yield of a Polymer OLED, Helping to Exceed the 25% Singlet Exciton Limit
Source: Adv Sci (Weinh). 2015 Dec 12;3(1):1500221. doi: 10.1002/advs.201500221 (PMC4991292; doi:10.1002/advs.201500221)
Supplement: Supplementary file 1 — Supplementary [file ADVS-3-0n-s001.pdf]

Copyright WILEY-VCH Verlag GmbH & Co. KGaA, 69469 Weinheim, Germany, 2015.

ADVANCED SCIENCE
Open Access

Supporting Information

for *Adv. Sci.*, DOI: 10.1002/advs. 201500221

# Generating Light from Upper Excited Triplet States: A Contribution to the Indirect Singlet Yield of a Polymer OLED, Helping to Exceed the 25% Singlet Exciton Limit

*Vygintas Jankus, Murat Aydemir, Fernando B. Dias, and Andrew P. Monkman**

# Supporting Information.

**Generating light from upper excited triplet states; a contribution to the indirect singlet yield of a polymer OLED, helping to exceed the 25% singlet exciton limit.**

*Vygintas Jankus, Murat Aydemir, Fernando B. Dias and Andrew P. Monkman**

*1. Possible triplet annihilation outcomes assuming quintuplets states are not accessible.*

Possible outcome after two triplets annihilate (ref 8):

$^3A^* + ^3A^* \rightarrow$

$1/9\ ^1(AA)^{**} \longrightarrow\ ^1A^* + ^1A_0$

$3/9\ ^3(AA)^{**} \longrightarrow\ ^3A^* + ^1A_0$

$5/9\ ^5(AA)^{**} \longrightarrow\ ^3A^* + ^1A_0$ or $^3A^* + ^3A^*$

Then assuming quintuplets are not accessible (ref 13) we get the following scheme:

$^5(AA)^{**} \rightarrow\ ^3A^* + ^1A_0$ or $^5(AA)^{**} \rightarrow\ ^3A^* + ^3A^*$

triplet recycling

*singlet per triplet yield*

$$\sum_{n=0}^{\infty} 8^n / 18^{n+1} = 0.1$$

*singlet per triplet yield*

$$\sum_{n=0}^{\infty} 13^n / 18^{n+1} = 0.2$$

| | |
|---|---|
| *EL yield: 0.25+(0.75x0.1)=0.325* | *EL yield: 0.25+(0.75x0.2)=0.4* |
| *DF gives 23% total EL* | *DF gives 37.5% total EL* |

*2. DF power dependence*

The DF decay (at 15 µJ per pulse excitation) displayed in Fig. 1 at 20 K approaches a power law with the slope -1. This type of decay at low temperatures has also been observed in other

organic films, namely, polyfluorene and *N*, *N*'-diphenyl-*N*, *N*'-bis(1-naphthyl)-1, 1'-biphenyl-4, 4''-diamine (NPB) and assigned to DF arising from triplet fusion. We refer to publications [3-6] for more detailed explanations of the physics of this type of power law dynamics.

*3. Delayed fluorescence yield calculations.*

The intensity ratio of DF to PF intensity can be defined as:

$$R = \frac{DF}{PF} = \frac{F_{inst} \cdot N_s \cdot \phi_f \cdot \phi_{DF} \cdot \phi_T + F_{inst} \cdot N_s \cdot \phi_f \cdot \phi_{DF}{}^2 \cdot \phi_T{}^2 + \ldots}{F_{inst} \cdot N_s \cdot \phi_f} = \frac{F_{inst} \cdot N_s \cdot \phi_f \cdot \left( \frac{\phi_T \cdot \phi_{DF}}{1 - \phi_T \cdot \phi_{DF}} \right)}{F_{inst} \cdot N_s \cdot \phi_f} \quad (1)$$

where $F_{inst}$ is an instrumental function, $N_s$ is number of excited states, $\varphi_f$ is fluorescence quantum yield, $\varphi_T$ is triplet yield and $\varphi_{DF}$ is DF yield from TF. Using our methodology, $F_{inst}$ is the same for PF and DF as they are recorded during the same measurement, likewise the initial number of excited states after pulsed excitation. All quenching mechanisms including intersystem crossing, non-radiative decay rate, quenching due to exciton migration are accounted for in $\varphi_f$ and are identical for both types of emission as again they come from same singlet state.

In order to avoid under evaluation of PF due to singlet-singlet annihilation [21] we have combined nanosecond gated time resolved spectroscopy (400 ps resolution) with single photon counting spectroscopy- TCSPC (3 ps resolution). The two measurements have good overlap to enable accurate data set combination (Figure 1b inset). No difference in the early time region of PF decay measured using gated nanosecond time resolved measurement (~from 1 ns to 20 ns) at low (1 µJ per pulse ) or high (15 µJ per pulse) excitation was found compared to TCSPC decay (<1nJ per pulse). 1 µJ per pulse is well within the measured linear excitation dependency regime (see Fig. 2a) indicating the absence of singlet-singlet annihilation effects, whereas <1nJ per pulse in TCSPC system is many orders of magnitude

less than singlet-singlet annihilation limit in polymers [21] . These combined curves are fitted with equation 2. Then solving for $\varphi_{DF}$ in equation 3 we obtain:

$$\phi_{DF} = \frac{R}{(1+R)\phi_T} \qquad (2)$$

To determine $\varphi_{DF}$ we evaluate R from the data in Fig. 1 by fitting equation (2) and taking appropriate DF and PF parts. PF integrals were calculated from 0 ns to ∞ and DF from 19 ns to 1s which leads to the average value of R=0.041±0.003. The start of the DF calculation is taken as the inverse value of the intersystem crossing rate of PSBF.[20] This means that we assume that DF starts when 67% of triplets have formed, such that the concentration is high enough to achieve TTA. $\varphi_T$=0.12±0.02 was taken from ref [20]. Using this information we arrive at the $\varphi_{DF}$ of 0.33±0.08. This value represents a lower limit of $\varphi_{DF}$ as it is impossible to account for all triplet exciton quenching during these measurements. For self-consistency, we also calculate the total emission of the decay curve by integrating the area under the curve and subtract the PF exponential part (as the exponent is a convergent function), yielding a comparable value of $\varphi_{DF}$ =0.32±0.08.
